# Underwater Image Enhancement Based on Luminance Reconstruction by Multi-Resolution Fusion of RGB Channels

**DOI:** 10.3390/s24175776

**Published:** 2024-09-05

**Authors:** Yi Wang, Zhihua Chen, Guoxu Yan, Jiarui Zhang, Bo Hu

**Affiliations:** National Key Laboratory of Transient Physics, Nanjing University of Science and Technology, Nanjing 210094, China; wangyi_zd@njust.edu.cn (Y.W.); ygx@njust.edu.cn (G.Y.); 121121011576@njust.edu.cn (J.Z.); hubo@njust.edu.cn (B.H.)

**Keywords:** underwater images, original image fusion, luminance reconstruction, multi-resolution fusion

## Abstract

Underwater image enhancement technology is crucial for the human exploration and exploitation of marine resources. The visibility of underwater images is affected by visible light attenuation. This paper proposes an image reconstruction method based on the decomposition–fusion of multi-channel luminance data to enhance the visibility of underwater images. The proposed method is a single-image approach to cope with the condition that underwater paired images are difficult to obtain. The original image is first divided into its three RGB channels. To reduce artifacts and inconsistencies in the fused images, a multi-resolution fusion process based on the Laplace–Gaussian pyramid guided by a weight map is employed. Image saliency analysis and mask sharpening methods are also introduced to color-correct the fused images. The results indicate that the method presented in this paper effectively enhances the visibility of dark regions in the original image and globally improves its color, contrast, and sharpness compared to current state-of-the-art methods. Our method can enhance underwater images in engineering practice, laying the foundation for in-depth research on underwater images.

## 1. Introduction

The oceans are rich in natural resources, and technological advancements have accelerated their exploration [1,2,3,4,5]. Underwater imaging is crucial for exploring and exploiting marine resources [2]. It has various applications, including detecting cracks in underwater dams [6], tracking fish [1,7], studying marine biodiversity studies [8,9,10], and conducting archeology [11].

**Figure 1:**
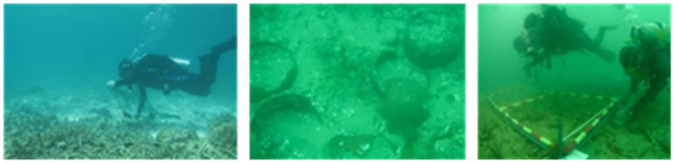
Examples of underwater images.

Compared to atmospheric imaging, underwater images are less clear due to the presence of suspended particles in the water, which causes light to scatter more [12,13,14,15]. Additionally, water is a ‘sticky’ substance that absorbs the energy of light, causing more refraction and altering the direction of light propagation in underwater imaging. Underwater, different wavelengths of electromagnetic waves attenuate differently [16,17]. For instance, blue and green light can reach farther regions than red light, resulting in the hue of underwater images being biased towards blue and green [12,18]. The imaging depth of underwater images is reduced by the complexity of the underwater situation, resulting in a foggy appearance and low contrast colors due to a large color bias [19]. This is illustrated in Figure 1.

To optimize the quality of images captured underwater, two principal methodologies have been employed: improving the imaging hardware and implementing software techniques [20] for image processing. Hardware techniques [21] are typically employed in the initial research phase, which includes enhancing the underwater imaging environment, utilizing polarization lenses [22], modifying sensors, and so on. Image-processing techniques have strong practical significance in underwater image recovery and enhancement due to their simplicity, cost-effectiveness, and ability to overcome environmental constraints in contrast to hardware methods which are more complicated, power-consuming, expensive, and vulnerable to such constraints [23,24].

**Figure 2:**
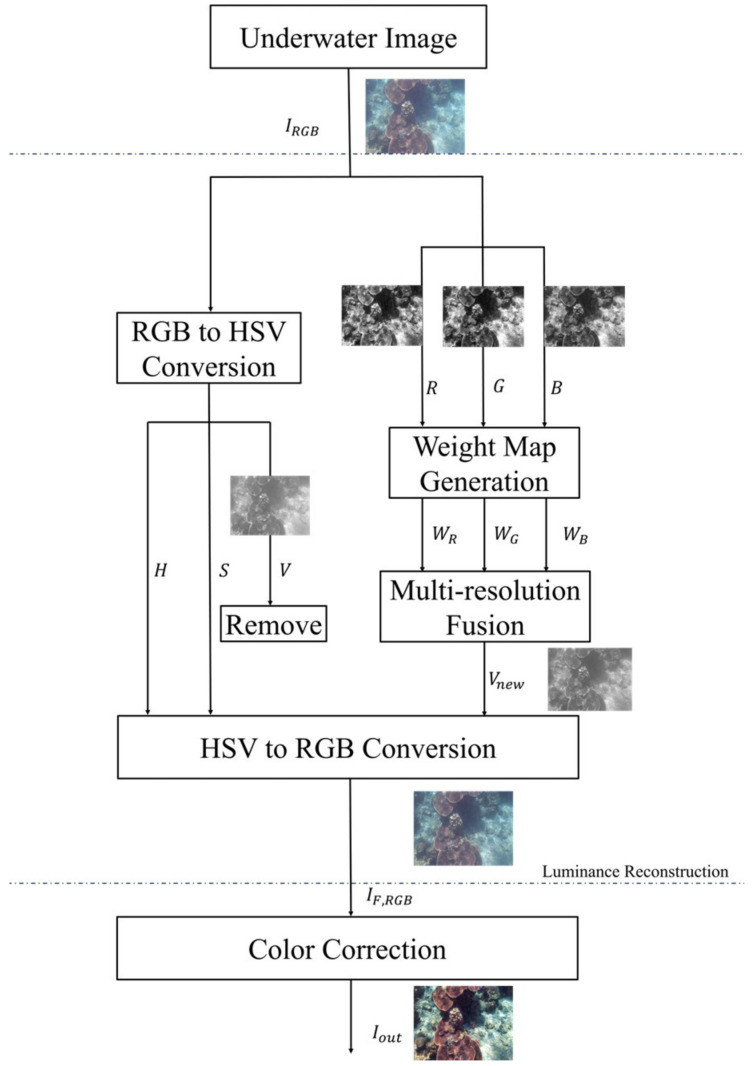
Workflow of our method.

Given the high cost of hardware and the difficulty of acquiring underwater image pairs, our objective is to develop a straightforward and computationally inexpensive method that can perform relatively fast operations on common hardware to enhance the visibility of underwater images. This paper proposes an image enhancement method for underwater images based on single optical image fusion. Unlike previous methods, our approach does not require complex transformations, as shown in Figure 2.

Contributions. This paper introduces the following main contributions:

(1) The hidden information of the image is excavated by considering the three RGB channels of the image as three images of different bands. These are used as the three inputs to reconstruct the image luminance by multi-resolution fusion. This method preserves the details of each input and enhances the visibility of the image.
(2) Enhanced white balance is employed for the initial color correction and sharpening of the image. We have improved the original white balance method of restoring the red channel; in addition, the blue or green channel is color-corrected according to the appearance of the image.
(3) Saliency detection techniques are applied to improve the hazy appearance of images. Saliency detection simulates human visual characteristics to make objects in the image stand out from the background, giving the underwater image a clearer visual effect.

The rest of the paper is structured as follows: Section 2 will present an overview of the current state-of-the-art development of key technologies for underwater image processing. Section 3 presents the method for reconstructing image luminance by fusing information from the three RGB channels. This includes generating the multi-resolution fusion weight map and adjusting the image color after luminance reconstruction. The following section presents a qualitative and quantitative comparison of the underwater image enhancement method proposed in this paper with the existing common underwater image enhancement methods.

## 2. Related Work

Various categorizations exist for underwater image enhancement methods. They can be classified into multi-image processing and single-image processing methods [25] based on the number of input images.

Multi-image processing methods include polarization imaging-based and multi-image scene recovery-based methods. The polarization imaging method aims to restore the original image by using multiple images acquired by rotating the camera’s polarizer at specific angles [26]. On the other hand, the method for recovering multi-image scenes involves constructing a digital map using multiple images captured from identical positions [27]. It should be noted that these methods do not apply to video acquisition. Additionally, the multi-image scene recovery methods are highly complex and not practical for general users.

Hence, the single-image-based underwater image-processing method has emerged as the main focus of the current research in the field of underwater image enhancement technology [12,17,25,28]. Researchers initially applied image dehazing and cloud occlusion reduction techniques from atmospheric image processing to enhance underwater images. This is because clouds and haze in the atmosphere refract, scatter, and attenuate visible light, thereby influencing the processing of underwater images. Expanding on these initial studies, methods for enhancing single underwater images have been developed to some extent. These methods can be broadly classified into three categories: physical model-based, non-physical model-based, and deep learning-based [29].

### 2.1. Physical Model-Based Methods

The physical model-based methods stem from an optical modeling perspective, considering underwater imaging as an inverse problem of optical imaging. These methods emphasize the utilization of models and prior knowledge to estimate the underwater image. The main workflow of these methods involves establishing a degradation physical model, parameter estimation, and model inversion.

**Figure 3:**
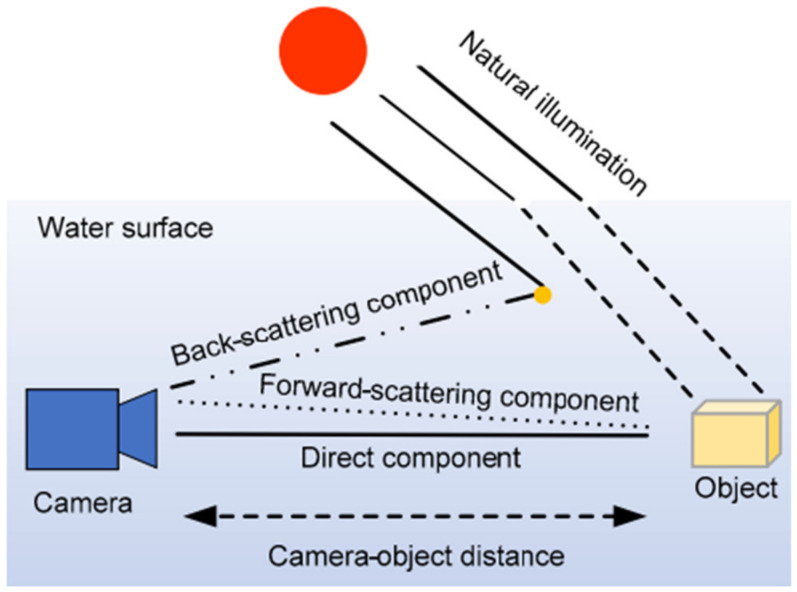
Schematic of the underwater optical imaging model. Reprinted from Xie et al. [31].

According to Jaffe–McGlamery’s underwater imaging model [30], as illustrated in Figure 3, the underwater optical signal received by the camera contains three components: direct component, forward scattering component, and backward scattering component.

The Jaffe–McGlamery model is currently the most widely used in underwater imaging [15]. Trucco et al. [32] proposed a self-adaptive underwater image restoration filter based on a simplified Jaffe–McGlamery model in 2006. This filter aimed to reduce the effect of light scattering in underwater images. In 2012, CHIANG et al. [17] introduced the Wavelength Compensation and Image Dehazing (WCID) method, which improved the luminance of images and mitigated the haze phenomenon.

Building upon the DCP model [33] for atmospheric dehazing, Drews et al. [34] proposed in 2013 an underwater dark channel prior (UDCP) specifically designed for underwater environments. This approach allowed for a more accurate estimation of the original image in underwater environments. In 2015, Galdran et al. [35] presented a method based on the red channel dark before addressing color attenuation issues in the red spectral band. In 2018, Tang et al. [36] proposed an improved dark channel prior image enhancement method. In this method, the luminance of the transmission map was enhanced using an adaptive scale selection strategy, and further color correction was performed to eliminate color deviations. In the same year, Peng et al. [37] improved the DCP-based method (GDCP), which can be applied to the enhancement and restoration of images affected by fog, haze, sandstorms, and underwater environments, including both well-lit and dimly lit images.

In 2020, Hou et al. [38] established an underwater total variation (UTV) model relying on underwater dark channel prior. The proposed method can achieve a good performance on dehazing, contrast enhancement, edge preservation, and noise suppression. In 2021, Xie et al. [39] introduced a red channel before the guided variational framework for underwater image dehazing and deblurring. The proposed UNTV method is based on the complete underwater image formation model including the forward scattering component. Li et al. [40] introduced a novel dual high-order regularization-guided variational framework (UDHTV) for underwater image restoration in 2024. In contrast to the majority of the extant model-based methodologies, which fail to account for the local illumination variations present in a single image, this approach constructs an extended underwater image model that incorporates these differences.

### 2.2. Non-Physical Model-Based Methods

Non-physical model-based methods for underwater image enhancement do not require the consideration of the underwater image model. Non-physical model-based methods for underwater image enhancement include histogram equalization, Retinex-based approaches, and image fusion techniques.

Histogram-based methods enhance images by stretching pixel values in one or multiple color spaces using various techniques. In 2010, Iqbal et al. [41] improved the color and contrast of underwater images by extending the dynamic pixel range in both the RGB and HSV color spaces. In the same year, Ghani et al. [42,43,44] further improved Iqbal’s method by implementing dynamic grayscale instead of dynamic pixels for image enhancement.

In 2017, Fu et al. [45] proposed a simple yet effective two-step enhancing strategy that requires only a single underwater image to be enhanced. Each step deals with one of the two major issues of color distortion and low contrast. For the color distortion problem, they proposed a color-correcting strategy based on a piecewise linear transformation.

The Retinex theory aims to restore illumination information in an image based on the characteristics of the human visual system. This approach involves multi-scale filtering to extract lightness and reflectance components at different scales, which are then adjusted to enhance the image. Retinex-based methods are effective in improving the luminance, contrast, and color of underwater images. Fu et al. [46] proposed a Bayesian Retinex approach that uses multi-scale gradient priors of reflectance and illumination to enhance single underwater images. Zhang et al. [47] also utilized an extended multi-scale Retinex method for enhancing underwater images. Additionally, Tang et al. [48] applied Retinex to enhance underwater videos.

For the enhancement of underwater images, fusion techniques can be used to merge images from different sensors or with different exposure or color characteristics. This improves image quality and visual perception. Ancuti et al. [49] developed a fusion-based model for underwater image enhancement. The model uses the results of histogram equalization to weigh the white balance color improvement and bilateral filtering output.

In 2022, Li et al. [50] propose a novel scheme by constructing an adaptive color and contrast enhancement, and denoising (ACCE-D) framework. In the proposed framework, the Difference of Gaussian (DoG) filter and the bilateral filter are employed to decompose the high-frequency and low-frequency components, respectively. In 2023, Zhang et al. [51] proposed a Piecewise Color Correction and Dual Prior Optimized Contrast Enhancement, called PCDE. They first considered the color cast of the underwater image. Afterward, they used different enhancement strategies to enhance the base and detail layers of the V channel in the HSV space decomposed using the spatial prior and texture prior.

Underwater image methods based on non-physical models aim to address various issues related to image quality degradation through a step-by-step approach. The enhancement effects of image quality are sequentially stacked in these methods, which have the advantages of high flexibility and the ability to be stacked. However, they often rely too heavily on pixel-level processing and neglect the principles of underwater optical imaging. This can result in enhanced images that do not accurately reflect the laws of underwater imaging.

### 2.3. Deep Learning-Based Methods

Deep learning technology has advanced significantly in recent decades and has been widely applied in computer vision and image-processing domains. Notably, the field of underwater image enhancement has made significant progress through the adoption of deep learning techniques [15,52,53]. As a result, many researchers have used deep learning approaches to improve the quality of underwater images [54].

In the early stages of research, convolutional neural networks (CNNs) emerged as a pioneering deep learning model and became one of the leading methods in the field [55]. When integrating CNNs with physical models for underwater image enhancement, the CNN model takes an underwater image as input and produces transmission maps and background light in the output layer. On the other hand, combining CNNs with non-physical models requires paired underwater images and their corresponding ground truth images. Importantly, the CNN model does not rely on prior knowledge, ensuring that the output images are unaffected by subjective factors.

Cai et al. [56] proposed a dehazing model based on the conventional convolutional neural network (CNN) architecture, which employed bilateral rectified linear units as activation functions. In 2018, Hou et al. [57] introduced residual learning into underwater image enhancement to enhance learning effectiveness. In 2020, Li et al. [58] constructed a benchmark dataset for underwater image enhancement using a large-scale collection of real underwater images. They further proposed an underwater image enhancement CNN trained on this dataset.

In recent years, there has been a growing trend toward the integration of generative adversarial networks (GANs) into machine learning methods [53,59,60,61,62]. Combining GANs with a physical model can effectively guide the synthesis of underwater images and facilitate the subsequent authenticity assessment through the discrimination process. This iterative procedure continues until the restored underwater image is obtained. Conversely, if GANs are combined with a non-physical model, the resulting underwater images are not limited by the constraints imposed by a physical model.

Fabbri et al. [63] proposed a method to enhance the visual quality of underwater images based on GANs, called the underwater generative adversarial network (UGAN). Wen Peizhi et al. [64] also developed a method for underwater image enhancement using GANs and improved convolutional neural networks (CNNs) to enhance the color correction capability of the models.

Current research faces a challenge in providing a substantial number of paired images for the effective training of deep learning-based methods for underwater image enhancement.

A variety of techniques have been investigated to improve the quality of underwater images. However, each approach has inherent limitations. The challenge for current researchers is to develop enhanced image-processing methods that require less computational power.

## 3. Proposed Algorithm

Figure 2 shows our proposed image enhancement method, which operates independently of the optical model of underwater images. It comprises two main stages: luminance reconstruction and color correction. In the luminance reconstruction stage, the initial image is decomposed into three separate single-channel images (red, green, and blue) using a multi-resolution fusion method to retain intricate details within the scene. Subsequently, the white balance color correction is improved by using a saliency map to compensate for color attenuation in underwater environments, resulting in overall image enhancement.

### 3.1. Decomposition of Image Color Space

In the dehazing methods for atmospheric images, researchers have incorporated near-infrared images to compensate for the attenuation caused by clouds and haze in the visible light spectrum. The near-infrared images are capable of penetrating clouds and haze, thereby enhancing the visibility of the atmospheric image [65]. This fusion technique has served as an inspiration for enhancing underwater images. However, it is important to note that not all images have paired visible light and near-infrared counterparts, as is the case with atmospheric images. Therefore, this study aims to explore the possibility of extracting relevant information directly from the original image that can produce a similar effect to fusing visible light and near-infrared images.

**Figure 4:**
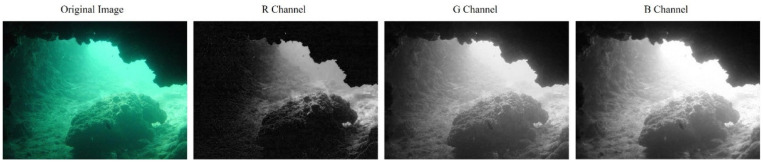
RGB spatial decomposition of the underwater image. From left to right: the original underwater image, the red channel image, the green channel image, and the blue channel image.

Different bands of visible light experience varying levels of attenuation underwater. The green band of visible light is relatively well preserved and retains a higher amount of image information. Underwater images are initially decomposed into the RGB color space, treating the R, G, and B channels as distinct channels representing different wavelengths of visible light. Figure 4 shows the original image of an underwater cave with noticeable red attenuation. The red channel provides more reliable information in regions of higher luminance, such as the upper part of the cave entrance. However, it fails to provide effective information in areas near the rocks due to the lower luminance levels. However, the original image loses some information near the cave entrance in the green and blue channels due to excessive luminance. In contrast, more details can be retained near the rocks.

This work aims to improve the quality of underwater images while preserving relevant information in each channel. The focus is on retaining more details, particularly related to luminance. To achieve this, we propose using the HSV color space, which effectively separates luminance from color and offers better color saturation preservation than other color spaces.

Expanding on the previous breakdown of color using the RGB color space, our next step involves rebuilding the brightness information within the HSV color space using the decomposed three-channel data obtained from the RGB color space. Subsequently, we apply color correction techniques to the rebuilt image to achieve the desired enhancement effect.

### 3.2. Multi-Resolution Fusion-Based Luminance Reconstruction

This section presents a new method for luminance reconstruction. First, we extract individual RGB channel images. Then, we use local entropy, local contrast, and local visibility measurements to create a weight map. This weight map guides the subsequent luminance reconstruction process, which involves the multi-resolution fusion of the single-channel images.

In image fusion, the main objective is to selectively retain the clearer and more detailed portions from each channel image. For instance, in Figure 4, we aim to preserve the red channel image information near the cave entrance and prioritize the blue and green channel information near the rocks. Suppose a simple weighted average fusion technique is used for all three channels. In that case, it may retain image information indiscriminately, regardless of whether there are significant details present in that particular area. As a result, this approach could blur crucial information and dilute prominent details.

To overcome these limitations, this research paper proposes using local entropy, local contrast, and local visibility measures to create a weight map. The weight map must meet the following criteria: weight values should range from 0 to 1, and the sum of the weight values across the three channels must be 1 for each pixel. This weight map enables a more precise selection of information during the fusion process, improving the overall quality and preserving the salient features in the final fused image.

The entropy of an image is a statistical metric that quantifies the information content of the image. It measures the average amount of information conveyed by the image. The entropy of a two-dimensional image is closely related to the amount of information contained in the distribution of gray levels within the image. This measure offers valuable information about the image’s spatial characteristics and clustering properties.

The proposal is to include a weighted measure, denoted as H, which quantifies the normalized local entropy. This measure is computed within the vicinity of a specific pixel (x,y) using the neighboring region N.

(1) $$H(x,y)=-\sum_{(i,j)\in N}p\left(f,\left(i,j\right)\right){log}_{2}p(f,(i,j))$$

The probability of observing the fth gray level is denoted by the symbol $p\left(f\right)$. In this implementation, a predetermined neighborhood with a size of 7 × 7 is employed. Therefore, to compute $p\left(f\right)$, the number of pixels with the same value as the pixel located at position $\left(i,j\right)$ within the 7 × 7 neighborhood centered around it, including the pixel itself, is tallied. The desired probability is obtained by dividing the count by the total number of pixels within the neighborhood.

To guarantee the accuracy of *H* in practical calculations, it is essential to apply the subsequent transformations to the *H* formula:

(2) $$H\left(x,y\right)=-\sum_{\left(i,j\right)\in N}p\left(f,\left(i,j\right)\right){log}_{2}\;num\left(f,\left(i,j\right)\right)+\sum_{(i,j)\in N}p\left(f,\left(i,j\right)\right){log}_{2}n\;$$

The equation defines num(f,(i,j) as the count of pixels in the 7 × 7 neighborhood centered at pixel $\left(i,j\right)$ that share the same value as the pixel $\left(i,j\right)$, including the pixel $\left(i,j\right)$ itself. Here, n represents the total number of pixels in the local neighborhood N. This approach ensures computational accuracy is not lost due to excessively small values of $p\left(f,\left(i,j\right)\right)$.

Local contrast is the difference in intensity between the target and the background within a given image or region of interest. Its main purpose is to enhance fine details while reducing the impact of the background. The local contrast coefficient (C) is calculated within a local neighborhood (N) centered around pixel coordinates $\left(x,y\right)$. It is defined mathematically as follows:

(3) $$\;\;\;C(x,y)=\sqrt{\frac{1}{n}\sum_{(i,j)\in N}(I\left(i,j\right)-\mu \left(x,y\right){)}^{2}}$$

$\mu \left(x,y\right)$ is defined as follows:

(4) $$\mu \left(x,y\right)=\sum_{(i,j)\in N}I\left(i,j\right)$$

where $I\left(i,j\right)$ represents the pixel value of the input image. Higher weights and contrasts are indicative of more intricate details within the region if the neighboring pixels exhibit greater deviations from the local mean. This paper uses a fixed neighborhood with dimensions of 7 × 7.

Local visibility is evaluated by assessing the channel’s ability to capture specific details within a given medium, such as water. A fuzzy estimation approach is employed using a Gaussian function with a predetermined standard deviation to induce blurring in the image, denoted as $I_{blur}$. The square root of the difference between the original image and its blurred counterpart is then used as the quantitative indicator of visibility, referred to as V.

(5) $$I_{blur}(x,y)=I(x,y)⛒g_{M}(x,y,\;\sigma 1)\;$$

(6) $$V(x,y)=\sqrt{{\left(I\left(x,y\right)-I_{blur}(x,y)\right)}^{2}⛒g_{M}(x,y,\;\sigma 2)}$$

The function $\;g_{M}(x,y,\;\sigma )\;$ represents a two-dimensional circular symmetric Gaussian weighted function with an M×M window and a standard deviation of σ2. The operator denotes a two-dimensional convolution. In this implementation, M is set to 7 and both σ1 and σ2 are set to 2.

The local entropy, local contrast, and local visibility weights are normalized and then combined through multiplication to derive a resultant weight. This study postulates that each of the three weights should have an equal influence on the final fusion outcome. Therefore, the weight assigned to each pixel can be represented as follows:

(7) $$W_{i}(x,y)=H_{i}(x,y)*C_{i}(x,y)*V_{i}(x,y)$$

where $i\in \left\{R,G,B\right\}$, denoting the three channels R, G, and B. Subsequently, we normalize the weights to ensure their summation is equal to 1 at each pixel location.

**Figure 5:**
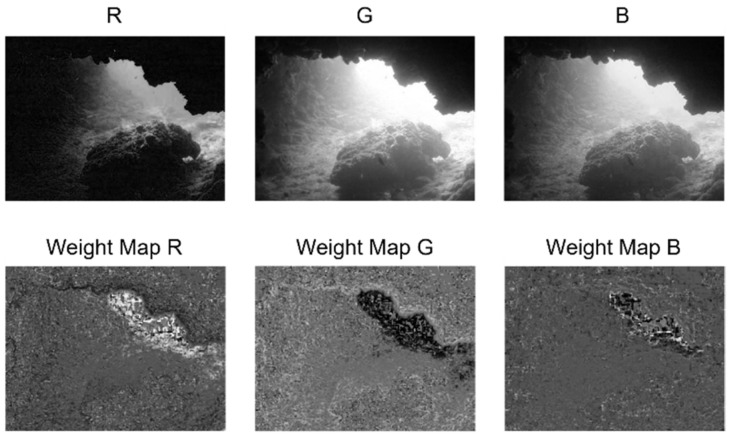
RGB channels of the underwater images and their weight maps. First row from left to right: image of the red channel, image of the green channel, and image of the blue channel; second row from left to right: weight maps generated for the red channel, green channel, and blue channel.

Figure 5 shows the resulting weight map generated for each channel of the underwater image shown in Figure 4. In this plot, a lighter weight map indicates a higher weight assigned to the corresponding point. As can be seen, the red channel, which is better preserved near the entrance to the cave, has a higher weight. Conversely, the blue and green channels have relatively higher weights near the rocks.

After obtaining the normalized weight maps, a multi-resolution fusion approach is applied to perform image fusion. In this section, we propose a technique that utilizes Laplacian pyramid decomposition for three single-channel images, Gaussian pyramid decomposition for the weight maps, and subsequently conducts fusion using Laplacian pyramid.

The lth level Gaussian pyramid decomposition of the weight map can be represented as follows:

(8) $${G\{W(x,y)\}}_{l}=[g(x,y)⛒{G\{W(x,y)\}}_{l-1}{]}_{↓2}$$

where g(x,y) represents the Gaussian kernel, ${G\{W(x,y)\}}_{l}\;$ denotes the approximation from the previous level, and ↓2 signifies the downsampling operation that reduces the area of the newly obtained image to one-fourth of the lower level. The Gaussian function exhibits separability, enabling us to simplify the algorithm implementation through the utilization of a 7 × 7 Gaussian filter.

The decomposition of the *l*-th level of the image using the Laplacian pyramid can be represented as follows:

(9) $${L\{I(x,y)\}}_{l}={G\{I(x,y)\}}_{l-1}-R\left(x,y\right)⛒{\left[{G\{I(x,y)\}}_{l}\right]}_{↑2}$$

where $R\left(x,y\right)$ signifies the interpolation filter, while the notation ↑2 denotes the upsampling operation by 2.

To construct the Laplacian pyramid for the given set of three input images, multiply each image’s corresponding level with the respective weight map and then sum them together. The reconstruction process follows the established Laplacian pyramid method, starting from the highest level and progressively adding it to the subsequent lower level until reaching the lowest level of the pyramid. The process can be defined as follows:

(10) $${L\left\{I_{F}\left(x,y\right)\right\}}_{l}={G\left\{W_{R}\left(x,y\right)\right\}}_{l}{L\left\{I_{R}\left(x,y\right)\right\}}_{l}+{G\left\{W_{G}\left(x,y\right)\right\}}_{l}{L\left\{I_{G}\left(x,y\right)\right\}}_{l}+{G\{W_{B}(x,y)\}}_{l}{L\{I_{B}(x,y)\}}_{l}$$

Finally, the reconstructed image $I_{F}$ is a matrix of identical dimensions to the source image. The pixel values of the $I_{F}$ image are then linearly scaled from the range of $\left[0,max(I_{F})\right]$ to the normalized range of [0, 1]. This normalization process effectively represents the reconstructed luminance component:

(11) $$V_{new}\left(x,y\right)=gamma\left(I_{F}\left(x,y\right),1\right)$$

$where\;gamma\left(I_{F}\left(x,y\right),1\right)$ is a correction function used for gamma adjustment. It applies a correction factor of 1.

### 3.3. Color Correction

After integrating luminance reconstruction, we apply color correction to the fused image using a method that combines the RGB and HSV color spaces. We begin with an enhanced white balance and saliency detection technique for preliminary color correction and image sharpening. Then, we rectify the hazy appearance of the image within the HSV color space, leveraging the specific properties of underwater images.

Following the luminance reconstruction, the image is restored from the HSV space to the RGB space. This process alters the overall color of the image due to the change in luminance. To correct the color, preliminary color correction is performed for each component (R, G, and B) of the restored image as outlined below:

(12) $$I_{T,RGB}(x,y)={\left(\frac{I_{F,RGB}(x,y)}{V_{new}\left(x,y\right)}\right)}^{\alpha }\left(\frac{I_{R}\left(x,y\right)+I_{G}\left(x,y\right)+I_{B}\left(x,y\right)}{3}\right)$$

where $I_{T,RGB}(x,y)\;$ represents the preliminary color-corrected image, $I_{F,RGB}\left(x,y\right)$ represents the fused RGB image, and α is the control parameter set to 1.5 as specified in this paper.

Next, we will perform color correction on underwater images. Our analysis of numerous underwater images has shown that the green and blue components remain intact, while the red component undergoes significant attenuation. Therefore, we have developed an enhanced white balance methodology based on the established criteria:

Compensating the red channel based on the blue and green channels. Compensation levels are determined by the pixel value of the red channel and the information conveyed by the blue and green channels. Lower compensation levels are required for pixels with higher values in the red channel or those with limited information in the blue and green channels. Conversely, Higher compensation levels are required for pixels with lower values in the red channel or those with abundant information in the blue and green channels.

In underwater images, when the blue component is excessively attenuated, compensation for the reduced blue channel is necessary by utilizing the green channel. This compensation method follows a similar principle as the approach used for the red channel. However, in this scenario, it is essential to artificially attenuate the green channel to achieve optimal results. Otherwise, if the blue component is high, artificial attenuation is applied to the blue channel.

Following the prescribed criteria, we will first establish the compensation formula for the red channel. It is important to note that before starting the calculation process, the pixel values of the RGB image must be normalized to fall within the range of [0, 1].

(13) $$I_{C,R}=I_{T,R}+\frac{1}{2}*\frac{\arg\left(I_{T,G}\right)-\arg\left(I_{T,R}\right)}{\arg\left(I_{T,R}\right)+\arg\left(I_{T,G}\right)+\arg\left(I_{T,B}\right)}*\left(1-I_{T,R}\right)*I_{T,G}+\frac{1}{2}*\frac{\arg\left(I_{T,B}\right)-\arg\left(I_{T,R}\right)}{\arg\left(I_{T,R}\right)+\arg\left(I_{T,G}\right)+\arg\left(I_{T,B}\right)}*\left(1-I_{T,R}\right)*I_{T,B}$$

where $I_{C,R}$ represents the corrected red channel. $\arg\left(I\right)$ represents the average pixel intensity of the image.

When $\arg\left(I_{T,G}\right)>\arg\left(I_{T,B}\right)$, it can be inferred that the attenuation of the blue component is higher. The compensation formula for the blue channel and the artificial attenuation formula for the green channel are provided below:

(14) $$I_{C,B}=I_{T,B}+\frac{\arg\left(I_{T,G}\right)-\arg\left(I_{T,B}\right)}{\arg\left(I_{T,R}\right)+\arg\left(I_{T,G}\right)+\arg\left(I_{T,B}\right)}*\left(1-I_{T,B}\right)*I_{T,G}$$

(15) $$I_{C,G}=I_{T,G}-\frac{\arg\left(I_{T,G}\right)-\arg\left(I_{T,B}\right)}{\arg\left(I_{T,R}\right)+\arg\left(I_{T,G}\right)+\arg\left(I_{T,B}\right)}*I_{T,G}$$

The formula for artificial attenuation of the blue component is as follows:

(16) $$I_{C,B}=I_{T,B}-\frac{\arg\left(I_{T,B}\right)-\arg\left(I_{T,G}\right)}{\arg\left(I_{T,R}\right)+\arg\left(I_{T,G}\right)+\arg\left(I_{T,B}\right)}*I_{T,B}$$

After compensating and attenuating pixels using artificial techniques, we integrated the saliency detection method proposed by Hou et al. [66] into our study. This method allows us to identify the most important areas within the image scene by analyzing the spectral residue of the image in the spectral domain. Our objective is to enhance the visually sensitive regions of underwater images by integrating the saliency detection method. The improved white balance image is represented based on the saliency map as follows:

(17) $$I_{wb}=I_{C}+saliencyMap*I_{C}$$

where saliencyMap refers to the saliency map. In our study, we only preserve the regions that exceed the automatically determined threshold.

We then use a non-masked sharpening technique to enhance the white-balanced image:

(18) $$I_{sharp}=I_{wb}+\frac{1}{4}Nor\left(I_{wb}-g⛒I_{wb}\right)$$

where $Nor\left(I\right)$ denotes the linear normalization of an image, while g represents a Gaussian filter.

Underwater images often appear hazy due to similar color values and reduced saturation in certain areas. To address this issue, we use histogram equalization to improve image sharpness and enhance local contrast. This technique results in a clearer and more visually appealing image. However, it is worth noting that histogram equalization can cause significant changes in color for certain images and may reduce the level of detail in specific regions. Therefore, we chose to convert the histogram-equalized and sharpened images to the HSV color space, integrate them with the initial reconstructed luminance, and then apply gamma correction to achieve the desired enhancement in the final image:

(19) $$H_{eh}=H_{sharp}$$

(20) $$S_{eh}=gamma\left({\lambda_{1}*S}_{sharp}+{\left(1-\lambda_{1}\right)S}_{histeq}\;,\alpha \right)$$

(21) $$V_{eh}=gamma\left({\lambda_{2}*V}_{sharp}+{\lambda_{3}*V}_{histeq}+{\left(1-\lambda_{2}-\lambda_{3}\right)I}_{F}\;,\;\;\beta \right)$$

In the HSV color space, $H_{sharp}$, $S_{sharp}$, and $V_{sharp}$ correspond to the three elements of the sharpened image. $S_{histeq}$ and $V_{histeq}$ represent the saturation and luminance components of the sharpened image following the histogram equalization. The correction coefficients are denoted by α and β, while $\lambda_{1}$, $\lambda_{2}$, and $\lambda_{3}$ signify the weighting factors. The values of $\lambda_{1}$, $\lambda_{2}$, and $\lambda_{3}$ must be within the range [0, 1], and the sum of $\lambda_{2}$, and $\lambda_{3}$ should also be within this range. Finally, the image is converted to the RGB color space, resulting in $I_{eh}$.

Finally, the image is processed based on the gray world principle [67]. To improve performance under underwater conditions, we employ the following adapted gray world method:

(22) $$f=1.2*min(max(\bar{I_{eh,R}},\bar{I_{eh,G}},\bar{I_{eh,B}}),0.375)$$

(23) $$I_{out,S}=\frac{{f*I}_{eh,S}}{\bar{I_{eh,S}}}$$

where S∈[R,G,B], $\bar{I_{eh,R}},\bar{I_{eh,G}},\bar{I_{eh,B}}$ represent the average values of the red, green, and blue channels, respectively, in the $I_{eh}$ image, and $I_{out}$ refers to the resultant final image.

## 4. Results and Discussion

In this section, we validated the methodologies presented in Section 3 and conducted a comparative analysis between our method and the existing methods for underwater enhancement. The experimental results in this paper are based on the underwater dataset UIEB proposed by Li et al. [58], and UCCS and UIQS proposed by Liu et al. [68]. UIEB includes 950 authentic underwater images, of which 890 have corresponding reference images, named standard dataset. Li et al. [58]’s method fails to produce satisfactory outcomes for the remaining 60 challenging dataset images. UCCS includes 300 images; this set aims to evaluate the ability of correcting color casts for algorithms. UIQS includes 3630 images; this subset is used to test algorithms for the improvement of image visibility.

To evaluate the effectiveness of the methods, this research paper will conduct a thorough analysis and comparison of the generated images under different environmental conditions using both objective evaluations and marked subjective evaluations. Firstly, we will assess the performance of various methods on the standard and challenging datasets separately while analyzing the outcomes of each method objectively.

### 4.1. Luminance Reconstruction Evaluation

**Figure 6:**
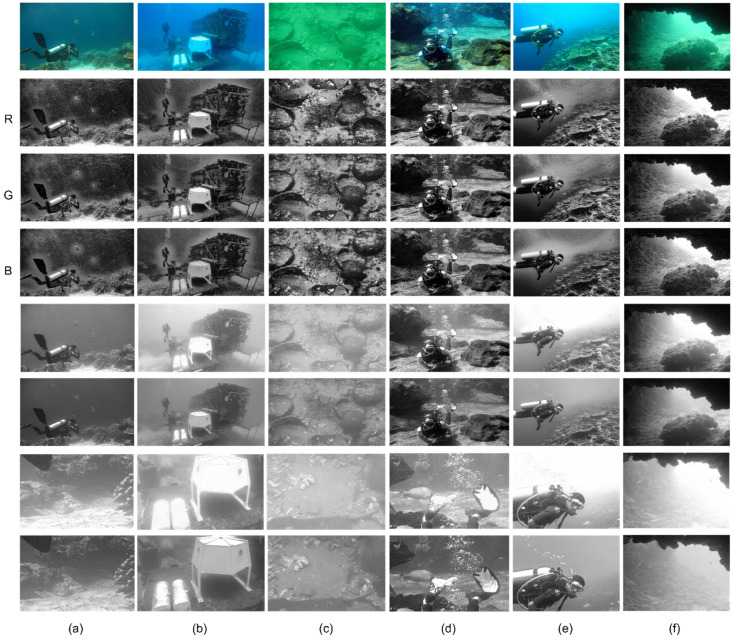
Example of luminance reconstruction results for part of the images from the image set. From left to right are six underwater images, from top to bottom: the original underwater images, the RGB components of the original images, the original luminance components of the images, the reconstructed luminance components, partially enlarged details of the original luminance components, and the corresponding enlarged details of the reconstructed luminance components. (**a**–**f**) are seven different underwater images.

Luminance reconstruction aims to preserve the most important information within each channel. This section conducts a qualitative analysis of the reconstructed luminance, focusing on its perceptual quality and fidelity. Figure 6 shows some examples of the luminance reconstruction results for part of the images from the image set.

As shown in Figure 6a, the original image displays excessive luminance in the zoomed-in area, leading to a loss of fine details near the coral. By reconstructing the luminance, the texture of the uneven ground surface becomes more prominent. In Figure 6b, several white objects underwater are affected by overexposure in the original luminance map, making it different to distinguish their specific contours. Luminance reconstruction highlights the corroded and rusted texture on the surface of the two cylindrical objects on the left, as well as the intersection lines between the faces of the polyhedron on the right.

Regarding Figure 6c, the original image has excessively high green channel values, resulting in an overall brighter luminance map. The reconstructed luminance enhances the contrast between the ground and the porcelain fragments, resulting in sharper edges of the fragments. In Figure 6d, the reconstructed luminance reveals the texture on the diver’s fins clearly, which was barely discernible in the previous image compared to the original luminance.

In Figure 6e, the background’s luminance near the original image’s water surface decreases significantly with depth, making it difficult to observe the bubbles exhaled by the diver. However, the reconstructed luminance map maintains a relatively consistent luminance across different underwater depths, making the bubbles stand out against the background.

The experimental results for Figure 6f are consistent with the hypothesis in section B of the third chapter: the reconstructed luminance map preserves more blue and green channel information near the rocks compared to the original luminance map while adding more red channel information near the cave entrance. As a result, the overall visibility of the image is improved.

### 4.2. Qualitative Evaluation

This section presents a comparative analysis between the method proposed in this paper and several established classical methods for underwater image enhancement. The parameters used for our method are specified as follows: $\lambda_{1}=0.6,{\;\lambda }_{2}=0.2,\;and\;\lambda_{3}=0.5;\;\alpha =0.85\;and\;\beta =1.5$.

The contrast methods under consideration for comparison consist of He et al.’s dark channel prior method (GDCP) [33], Peng et al.’s GDCP method [37], Fu et al.’s two-step method [45], Ancuti et al.’s fusion-based method [49], Hou et al.’s UVT method [38], Xie et al.’s UNVT method [39], and Zhang et al.’s PCDE method [51].

**Figure 7:**
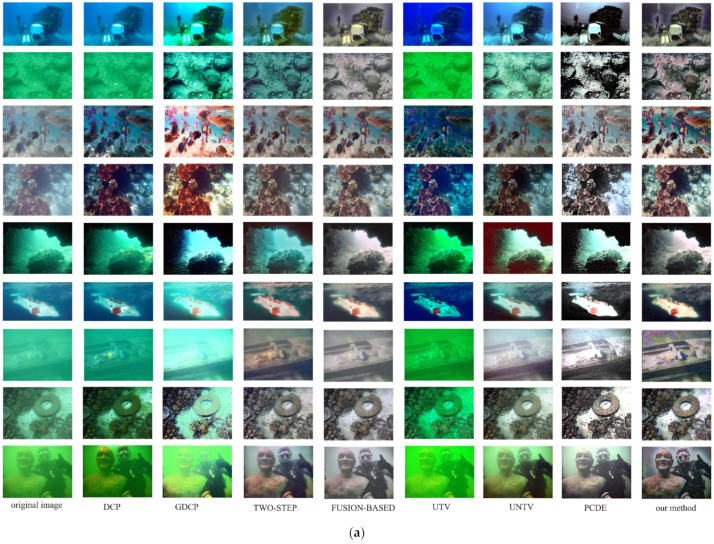

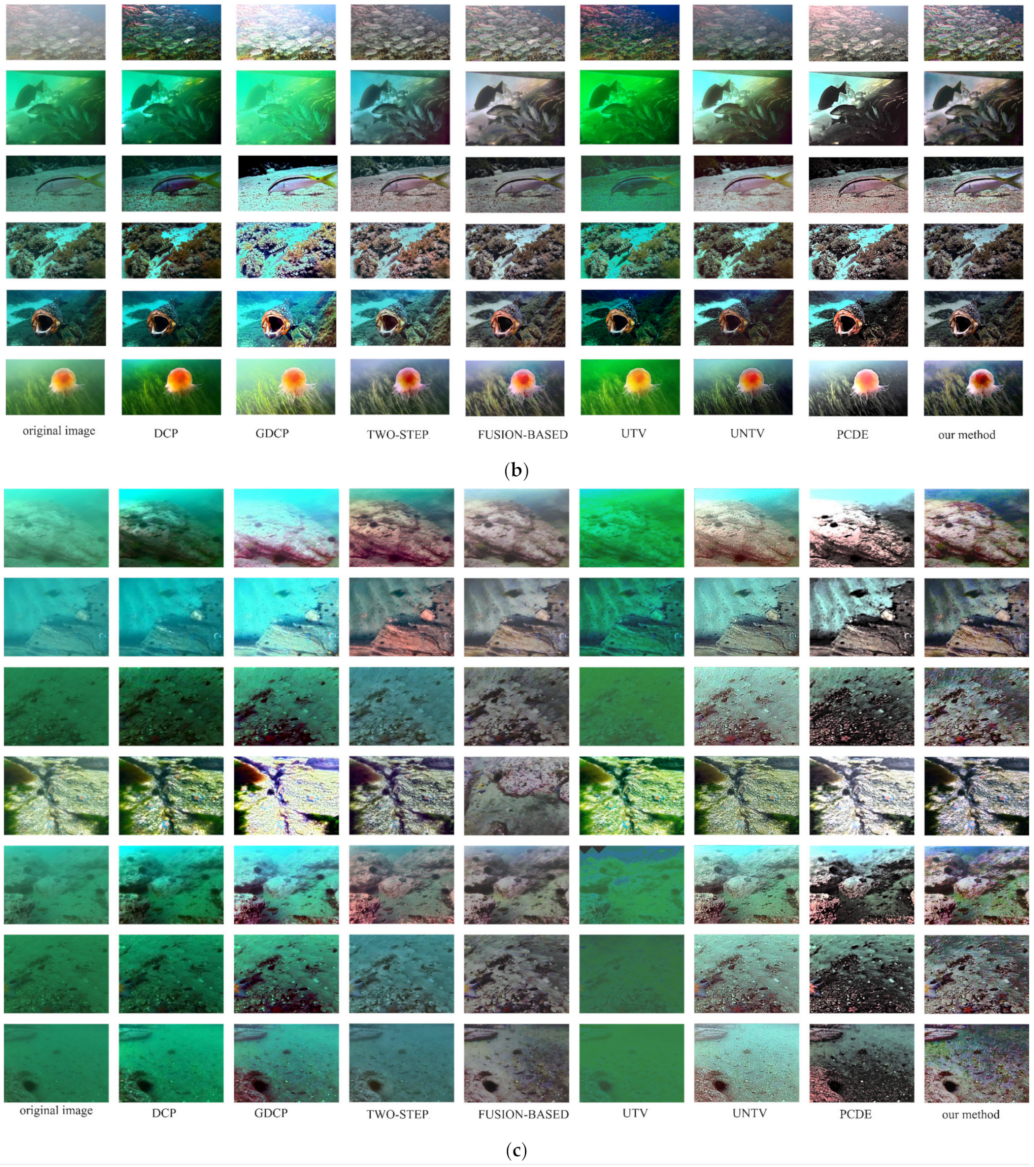
Examples of the results of different methods on underwater images. (**a**) Illustrative results of algorithmic analysis on the UIEB standard dataset; (**b**) illustrative results of algorithmic analysis on the UIEB challenging dataset; (**c**) illustrative results of algorithmic analysis on UCCS and UIQS dataset. From left to right: the original underwater image, results using DCP [33], GDCP [37], two-step [45], fusion-based [49], UTV [38], UNTV [39], PCDE [51], and our method.

Figure 7 shows some results of the different methods on underwater images.

Figure 7 shows that DCP [33], GDCP [37], and UTV [38] methods are not particularly effective at handling blue and green colors in the background of images. The results produced by GDCP are much better than DCP’s, but GDCP [37] may lead to overexposure in areas of the original image with high luminance levels. The two-step [45] method is incapable of processing the blue background present in the image, resulting in unstable performance. The UNTV [39] and PCDE [51] methods are superior to the methods mentioned above, nevertheless, the objects in the processed image still exhibit hues characteristic of blue or green. The images obtained by the PCDE method conceal a considerable amount of detail, both in bright and dark areas. Ancuti et al. [49]’s fusion-based method and the method proposed in this paper perform exemplary jobs of correcting the image’s color. However, the fusion-based method is conducive to a certain degree of haze-like appearance. The method proposed in this paper facilitates the restoration of image color in the presence of turbid water and non-uniform lighting conditions, while also enhancing image details. This is in contrast to several methods that have been mentioned above. Moreover, as shown in the first and ninth rows of Figure 7a, it can enhance foreground images while maintaining the background.

**Figure 8:**
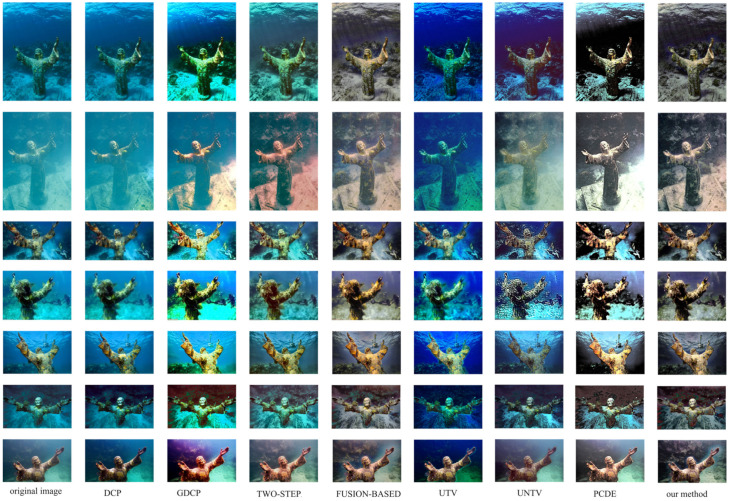
Results of different methods on underwater images from different angles in the same scene. From left to right: the original underwater image, results using DCP [33], GDCP [37], two-step [45], fusion-based [49], UTV [38], UNTV [39], PCDE [51], and our method.

Figure 8 illustrates the processing outcomes achieved by the methods on underwater images acquired from different perspectives of the identical scene. Consistent color representation should ideally be observed upon processing the same object. However, as depicted in Figure 8, the results using DCP [33], GDCP [37], two-step [45], UTV [38], UNTV [39], and PCDE [51] do not exhibit uniform colors for the restored sculptures. Conversely, the fusion-based [49] method and the method presented in this paper effectively restore objects in underwater images while maintaining similar color schemes. Additionally, the background colors produced by our method also demonstrate a remarkable level of cohesion.

### 4.3. Quantitative Evaluation

The method’s image quality was comprehensively evaluated using three metrics: UIQM [69] (Underwater Image Quality Measures), UCIQE [70] (Underwater Color Image Quality Evaluation Metric), FDUM [71] (Frequency Domain-based UIQA Method), CSN [72] (Contrast Sharpness and Naturalness), and image entropy.

Panetta et al. [69] proposed UIQM, a non-reference measure designed to assess the quality of underwater images. UIQM includes three attribute measurements inspired by the characteristics of the human visual system: color, sharpness, and contrast. the UIQM of a given image is calculated as follows:

(24) $$UIQM=c_{1}\times UICM+c_{2}\times UISM+c_{3}\times UIConM,$$

where UICM denotes the colorfulness, UISM denotes sharpness, and UIConM denotes the contrast; $c_{1}$, $c_{2}$, and $c_{3}\;$ are three weighted coefficients.

UCIQE, on the other hand, evaluates the quality of underwater color images by considering a weighted combination of chroma, saturation, and sharpness in the CIELab color space. The resulting UCIQE index ranges from 0 to 1, with higher values indicating better balance performance among chroma, saturation, and sharpness. A higher UCIQE score corresponds to a higher-quality underwater image. The definition of UCIQE is given by the following:

(25) $$UCIQE=c_{1}\times \sigma_{C}+c_{2}\times {con}_{L}+c_{3}\times \mu_{S}$$

where $\sigma_{C}$, ${con}_{L},$ and $\mu_{S}$ indicate the standard deviation of chroma, the contrast of luminance, and the average of saturation, respectively; $c_{1}$, $c_{2},$ and $c_{3}\;$ are three weighted coefficients.

FDUM is obtained by fusing the colorfulness, contrast, and sharpness measures. The formula is as follows:

(26) $$FDUM=\omega_{1}\times Colorfulness+\omega_{2}\times Contrast+\omega_{3}\times Sharpness$$

where $\omega_{1}$, $\omega_{2}$ and $\omega_{3}$ are the weighted coefficients corresponding to the colorfulness, contrast, and sharpness measurements, respectively.

CSN is a no-reference underwater IQA method which is designed by combining the contrast index, the sharpness index, and the naturalness statistical model.

Additionally, image entropy indicates the information content in an image. The image entropy metric measures the randomness or uncertainty present in the image data. Higher levels of entropy indicate a greater amount of information, while lower levels suggest a more predictable and less complex image.

Therefore, the UIQM, UCIQE, PDUM, and CSN evaluation metrics, along with image entropy, provide a comprehensive and professional assessment of the image quality of the method, taking into account various aspects such as color, sharpness, contrast, and information content.

Table 1 presents the evaluation metrics, namely UIQM, UCIQE, PDUM, CSN, and image entropy, for the methods applied to the UIEB standard set, UIEB challenging set, UCCS, and UIQS. These metrics reflect the average evaluation values across all the images within each dataset.

Our method achieves the top 2 scores for both UIQM and entropy on the four datasets, and both UIQM and entropy scored three top 1. In terms of the CSN metrics, our method achieved the highest score in two sets, came second in one, and was placed third in another.

It is interesting to note that the method introduced by He et al. [33] achieves the highest average UIQM score on the UIEB challenging set, which is intriguing. We conducted a thorough analysis of this matter and found that a majority of the 60 images included in the challenging dataset exhibit a hazy appearance. It should be noted that He et al.’s method [33] performs exceptionally well in haze removal, which is supported by both our analysis and the subjective evaluation of He et al.’s [33] defogging capabilities.

The UCIQE metric shows that Peng et al.’s GDCP method [37] and He et al.’s method [33] both achieved the top 2 UCIQE values on the four sets. However, our method’s performance ranked only in the middle, which contradicts our previous analysis. To further investigate this discrepancy, we conducted a series of experiments and made an interesting observation that the UCIQE method exhibits a bias toward image saturation. It was observed that increasing the saturation of certain images with pronounced color deviation, such as multiple images in the second and fifth rows of Figure 7a, resulted in a dominant green or blue appearance in the entire image. This led to a significant decrease in visibility, but an increase in the UCIQE values. Some of the evaluated methods with UCIQE values greater than 0.5 increased image saturation without implementing the proper color correction techniques. As a result, these images experienced color deviation and degraded visual effects, but still achieved higher UCIQE values.

To ensure a fair comparison of the UCIQE values, the methods lacking proper color correction should be excluded. After excluding such methods, our proposed method demonstrated an excellent UCIQE performance in this study.

It seems that our method did not achieve the top two positions in PDUM in Table 1. However, our method is currently ranked third, with a relatively narrow margin between it and the top two. Same as UCIQE, our method demonstrates favorable PDUM performance, with the exception of those algorithms that are unable to achieve superior color correction.

In addition, it can be seen that, except for the UIEB challenge set, which is an artificially selected image with particularly harsh underwater environments, the UIQM, UCIQE, and entropy results of this paper’s method on the UIEB standard set, the UCCS, and the UIQS, are virtually unchanged, which proves the stability of the method in this paper.

In light of the aforementioned methods, our method demonstrates superior efficacy in background color correction when compared to DCP [33], GDCP [37], and UTV [38]. Our method is efficacious in the removal of blue and green hues from the background. Compared to two-step [45], fusion-based [49], UNTV [39], and PCDE [51] methods, the method proposed in this paper is a more consistent means of removing the blue and green color from both the background and the objects therein. Furthermore, it maintains the vividness of the image’s color while retaining more detailed information and achieving favorable results in quantitative analysis.

## 5. Conclusions

This paper proposes a new method for enhancing underwater images using multi-resolution fusion for luminance reconstruction. The method aims to extract latent information from individual images by exploiting the RGB channels of the original image and subsequently performing color restoration. Extensive experimentation has shown that the method greatly improves image sharpness by emphasizing the importance of details within each channel. Furthermore, our color correction method effectively addresses color deviations caused by uneven underwater lighting conditions. As a result, our proposed method has significant potential for practical applications in engineering fields related to underwater image enhancement.

Our method also has several limitations. The multi-resolution fusion and Laplace–Gaussian pyramid approach may entail a considerable computational burden, rendering them unsuitable for real-time processing or deployment on lower-end devices. In future research, we will attempt to implement algorithmic lightweight. Furthermore, the technique of underwater image enhancement will be applied to other areas of research, including object recognition.

## Figures and Tables

**Table 1:** Underwater image enhancement evaluation based on UIQM, UCIQE, FDUM, CSN, and entropy metrics on UIEB standard set, UIEB challenging set, UCCS, and UIQS. The larger the metric the better.

**Methods**		**UIEB Standard Set**					**UIEB Challenging Set**			
	**UIQM**	**UCIQE**	**FDUM**	**CSN**	**Entropy**	**UIQM**	**UCIQE**	**FDUM**	**CSN**	**Entropy**
DCP [33]	2.955	0.579	0.664	0.581	7.267	3.943	0.575	0.506	0.557	6.780
GDCP [37]	2.652	0.604	0.866	0.599	7.195	1.479	0.569	0.661	0.586	7.383
Two-step [45]	3.779	0.488	0.619	0.551	7.457	2.134	0.476	0.397	0.495	7.215
Fusion-based [49]	4.219	0.450	0.520	0.548	7.419	2.724	0.427	0.327	0.484	7.148
UTV [38]	2.307	0.574	0.608	0.574	6.112	1.105	0.526	0.357	0.494	5.313
UNTV [39]	3.412	0.536	0.882	0.588	0.744	2.421	0.511	0.570	0.570	7.048
PCDE [51]	4.936	0.506	0.447	0.510	7.719	2.520	0.486	0.339	0.605	6.893
Our method	4.962	0.497	0.772	0.591	7.65 5	3.330	0.477	0.440	0.550	7.393
**Methods**		**UCCS**					**UIQS**			
	**UIQM**	**UCIQE**	**FDUM**	**CSN**	**Entropy**	**UIQM**	**UCIQE**	**FDUM**	**CSN**	**Entropy**
DCP [33]	1.656	0.579	0.453	0.432	7.320	1.980	0.577	0.489	0.522	7.290
GDCP [37]	4.743	0.563	0.457	0.474	7.539	4.824	0.565	0.501	0.469	7.565
Two-step [45]	3.687	0.437	0.457	0.450	7.230	3.808	0.439	0.447	0.444	7.245
Fusion-based [49]	3.723	0.405	0.313	0.422	7.184	3.860	0.415	0.333	0.515	7.230
UTV [38]	−0.376	0.514	0.248	0.371	5.294	−0.052	0.504	0.246	0.364	5.580
UNTV [39]	3.888	0.505	0.748	0.494	7.639	3.970	0.513	0.752	0.484	7.580
PCDE [51]	3.925	0.505	0.701	0.502	7.572	3.941	0.504	0.707	0.503	7.527
Our method	4.975	0.491	0.674	0.535	7.764	4.841	0.493	0.703	0.537	7.719

The most optimal result is red while the second best is blue. Values in the table are dimensionless.

## Data Availability

The original contributions presented in the study are included in the article, further inquiries can be directed to the corresponding author.
